# Two new aliphatic lactones from the fruits of *Coriandrum sativum* L.

**DOI:** 10.1186/2191-2858-2-28

**Published:** 2012-07-16

**Authors:** Kamran J Naquvi, Mohammed Ali, Javed Ahmad

**Affiliations:** 1 Department of Pharmacognosy and Phytochemistry, Faculty of Pharmacy, Jamia Hamdard, New Delhi, 110062, India

**Keywords:** *Coriandrum sativum*, Apiaceae, Aliphatic *δ*-lactones, Fatty acid glycerides

## Abstract

**Background:**

The present paper describes the isolation and characterization of two new aliphatic *δ*-lactones along with three glycerides and n-nonadecanyl cetoleate from the fruits of *Coriandrum sativum* L. (Apiaceae). The structures of all the isolated phytoconstituents have been established on the basis of spectral data analysis and chemical reactions.

**Results:**

Phytochemical investigation of the methanolic extract of *C. sativum* L. (Apiaceae) fruits resulted in the isolation of two new aliphatic *δ*-lactones characterized as 2α-n-heptatriacont-(Z)-3-en-1,5-olide (**1**) (coriander lactone) and 2α-n-tetracont-(Z,Z)-3,26-dien-18α-ol-1,5-olide (**2**) (hydroxy coriander lactone) together with glyceryl-1,2-dioctadec-9,12-dienoate-3-octadec-9-enoate (**3**); glyceryl-1,2,3-trioctadecanoate (**4**); n-nonadecanyl-n-docos-11-enoate (**5**) and oleiyl glucoside (**6**).

**Conclusions:**

Phytochemical investigation of the methanolic extract of *C. sativum* gave coriander lactone and hydroxy coriander lactone as the new phytoconstituents.

## Background

*Coriandrum sativum* L. is an annual and herbaceous plant belonging to the Apiaceae family. It is a medicinal plant, native of southern Europe and western Mediterranean region and is cultivated worldwide [1]. India is the largest producer of coriander in the world. Major production centers are Rajasthan, Maharastra, Gujarat, and Karnataka [2]. The whole plant and especially the unripe fruits are characterized by a strong disagreeable odor, hence the name coriander, giving characteristic aroma when rubbed [3]. The most important constituents of coriander seeds are the essential oil and the fatty oil. The dried coriander seeds contain an essential oil (0.03% to 2.6%) with linalool as main component [4,5], phenolics, flavonoides [6], and isocoumarin compounds [7]. It has traditionally been referred to as antidiabetic [8] and cholesterol lowering drug [9,10]. This paper describes the isolation and characterization of two new aliphatic *δ*-lactones along with fatty acid glycerides, ester, and glucoside from the fruits of *C. sativum* of Delhi region of India.

## Methods

### General

Melting points were determined on a Perfit melting apparatus (Ambala, Haryana, and India) and are uncorrected. UV spectra were measured with a Lambda Bio 20 spectrophotometer (Perkin-Elmer-Rotkreuz, Switzerland) in methanol. Infrared spectra were recorded on Bio-Rad FTIR 5000 (FTS 135, Kowloon, Hong Kong) spectrophotometer using KBr pellets; *γ*_max_ values are given in cm^−1^. ^1^ H nuclear magnetic resonance (NMR) and ^13^ C NMR spectra were screened on Bruker spectrospin 300 and 75 MHz, respectively, instruments (Karlesruthe, Germany) using CDCl_3_ and TMS as an internal standard. Mass spectra were measured by effecting fast atom bombardment (FAB) ionization at 70 eV on a JEOL-JMS-DX 303 spectrometer (JEOL, Japan) equipped with direct inlet probe system. Column chromatography was performed on silica gel (60 to 120 mesh; Qualigen, Mumbai, India). TLC was run on silica gel G (Qualigen, Carlsbad, CA). Spots were visualized by exposing to iodine vapors, UV radiation, and spraying with ceric sulphate.

## Results and discussion

Compound 1, named coriander lactone (Scheme 1), was obtained as a pale yellow crystalline mass from petroleum ether eluents. Its IR spectrum showed characteristic absorption bands for *δ*-lactone group (1738 cm^−1^), unsaturation (1621 cm^−1^), and long aliphatic chain (806, 721 cm^−1^). On the basis of FAB mass spectrum, its molecular weight was established at *m/z* 546 consistent with the molecular formula of an unsaturated lactone, C_37_ H_70_ O_2_. It indicated three double bond equivalents; two of them were adjusted in the lactone ring and remaining one in the vinylic linkage. The prominent ion peaks arising at *m/z* 97 [C_2_-C_6_ fission, C_5_H_5_O_2_]^+^ and 449 [M-97, (CH_2_)_31_ CH_3_]^+^ suggested that *δ* lactone was attached to the C-32 aliphatic chain. The ^1^ H NMR spectrum of **1** showed a one-proton double doublet at *δ* 5.36 (*J* = 6.6, 7.2 Hz) and a one-proton multiplet at *δ* 5.32 assigned to (Z)-vinylic H-3 and H-4 protons, respectively. A two-proton doublet at *δ* 4.14 (*J* = 7.2 Hz) was ascribed to oxygenated methylene H_2_-5 protons. A one-proton broad multiplet at *δ* 2.78 with half width of 6.1 Hz was attributed to β-oriented H-2 methine proton. A two-proton multiplet at *δ* 2.30, two four-proton multiplets at *δ* 2.05 and 1.59, and a broad signal at *δ* 1.26 integrating for 52 protons were associated with the methylene protons. A three-proton triplet at *δ* 0.90 (*J* = 6.3) was accounted to C-37 primary methyl protons. The ^13^ C NMR spectrum of **1** displayed signals for lactone carbon at *δ* 173.56 (C-1), vinylic carbons at *δ* 130.15 (C-3) and 127.91 (C-4), oxygenated methylene carbon *δ* 66.83 (C-5), methine carbon at *δ* 34.21 (C-2), methylene carbons between *δ* 31.91 and 22.64, and methyl carbon at *δ* 14.03 (C-37). The ^1^ H-^1^ H correlation spectroscopy (COSY) spectrum of **1** showed correlations of H_2_-5 with H-4 and H-3; and H-2 with H-3, H-4 and H_2_-6. The heteronuclear multiple bond correlation (HMBC) spectrum of **1** exhibited interactions of C-1 with H-2, H-3 and H_2_-6; and C-5 with H-4 and H-3. On the basis of the foregoing account, the structure of **1** has been established as 2-α-n-heptatriacont-(Z)-3-en-1,5-olide. This is a new *δ*-lactone isolated from a plant source [see Additional file 1].

Compound **2**, named hydroxy coriander lactone, was obtained as a pale yellow crystalline mass from chloroform eluents (Scheme 2). Its IR spectrum showed distinctive absorption bands for a hydroxy group (3463 cm^−1^), lactone ring (1743 cm^−1^), unsaturation (1645 cm^−1^), and long aliphatic chain (722 cm^−1^). Its FAB mass spectrum had a molecular ion peak at *m/z* 603 [M + H]^+^ corresponding to a long chain hydroxylated aliphatic lactone, C_40_H_75_O_3_. It showed four degrees of unsaturation; two each of them were adjusted in the lactone ring and two in vinylic linkages. The prominent ion peaks arising at *m/z* 97 [C_2_-C_6_ fission, C_5_H_5_O_2_]^+^, 265 [C_17_-C_18_ fission, C_5_H_5_O_2_(CH_2_)_12_]_,_^+^ 337 [M-265, HOCH(CH_2_)_7_CH = CH(CH_2_)_12_CH_3_]^+^, 295[C_18_-C_19_ fission, C_5_H_5_O_2_(CH_2_)_12_CHOH]^+^ suggested the presence of one of the vinylic linkage in the lactone ring and the hydroxyl group at C-18. The ion peak forming at *m/z* 393 [C_25_-C_26_ fission, C_5_H_5_O_2_(CH_2_)_12_CHOH(CH_2_)_7_]^+^ indicated the location of another vinylic linkage at C-26. The ^1^ H NMR spectrum of **2** showed a one-proton double doublet at *δ* 5.34 (6.8, 7.5 Hz) and four multiplets at 5.26, 5.17 (w_1/2_ = 8.5 Hz), and 5.11 (w_1/2_ = 9.5 Hz) assigned correspondingly to *cis*-oriented H-4, H-3, H-26, and H-27 vinylic protons, respectively. Two one-proton doublets at *δ* 4.32 (*J* = 7.5 Hz) and 4.28 (*J* = 7.5 Hz) were attributed to the oxygenated methylene H_2_-5. A one-proton broad multiplets at *δ* 4.16 with half width of 5.7 Hz was ascribed to β-oriented H-18 carbinol proton. A one-proton multiplet at *δ* 2.76 (w_1/2_ = 6.8 Hz) was accounted to β-oriented H-2 methine protons. Two multiplets at *δ* 2.30 and 2.02 integrating for two protons each were due to methylene H_2_-25 and H_2_-28 protons located nearby the C-26 and C-27 vinylic carbons. The remaining methylene protons resonated between *δ* 1.98 and 1.26. A three-proton triplet at *δ* 0.87 (*J* = 6.5 Hz) was associated with the C-40 primary methyl protons. The ^13^ C NMR spectrum of **2** displayed signals for lactone carbon at *δ* 172.78 (C-1), vinylic carbons at *δ* 130.34 (C-4), 129.78 (C-3), 128.78 (C-26), and 127.96 (C-27), carbinol carbon at *δ* 68.83 (C-18), oxygenated methylene carbon at *δ* 65.95 (C-5), methine carbon at *δ* 42.03 (C-2), methylene carbons between *δ* 33.76 and 22.55, and methyl carbon at *δ* 13.94 (C-40). The ^1^ H-^1^ H COSY spectrum of **2** showed correlations of H_2_-5 with H-4 and H-3; H-2 with H-3, H-4, and H_2_-6; H-18 with H_2_-17 and H_2_-19; H-26 with H_2_-25, H-27, and H_2_-28; and H_3_-40 with H_2_-39. The HMBC spectrum of **2** exhibited interactions of C-1 with H-2; C-3 with H-2, H_2_-6, and H-4; C-4 with H_2_-5 and H-3; C-18 with H_2_-17 and H_2_-19; and C-26 with H-27 and H_2_-25. These evidences led to formulate the structure of **2** as 2α-n-tetracont-(Z,Z)-3, 26-dien-18α-ol-1,5-olide. This is a new *δ*-lactone isolated from a plant source.

Earlier *δ*-lactonic constituents have been isolated from the root bark of *Capparis deciduas*[11], hulls of *Oryza sativa*[12], and seeds of *Althea officinalis*[13]. The compound 3 to 6 were the known phytoconstituents identified as glyceryl-1,2-dioctadec-9,12-dienoate-3-octadec-9,12-dienoate, glyceryl-1,2,3-trioctadecanoate, n-nonadecanyl-n-docos-1-enoate, and n-octadec-9-enyl-β-D-glucopyranoside, respectively.

## Experimental

### Plant material

The fruits of *C. sativum* were collected from the herbal garden of Jamia Hamdard, New Delhi. The plant material was identified by Prof. MP Sharma, Taxonomist, Department of Botany, Faculty of Science, Jamia Hamdard, New Delhi. A voucher specimen (PRL/JH/07/27) of drug is preserved in the Phytochemistry Research Laboratory, Department of Pharmacognosy and Phytochemistry, Faculty of Pharmacy, Jamia Hamdard, New Delhi.

### Extraction

*C. sativum* fruits (1.6 kg) were dried in air and then in an oven at 45°C temperature. The material was coarsely powdered. Exhaustive extraction of powdered drug was carried out in a Soxhlet apparatus using methanol as extracting solvent. The methanolic extract was concentrated under reduced pressure to yield a dark brown viscous mass, 187 g (11.68%).

### Isolation of phytoconstituents

The methanolic extract (85 g) was dissolved in a minimum amount of methanol and adsorbed on silica gel (60 to 120 mesh) for preparation of slurry. The air-dried slurry was chromatographed over the silica gel column packed in petroleum ether (60 to 80°C). The column was eluted with petroleum ether (60 to 80°C), chloroform, and methanol in order of increasing polarity to isolate the following compounds:

#### Coriander lactone (1)

Elution of the column with petroleum ether furnished pale yellow crystals of **1,** recrystallized from acetone, 76 mg (0.089% yield); *R*_f_ value, 0.9 (petroleum ether); m.p, 110 to 111°C; UV *λ*_max_ (MeOH), 206 nm (log ϵ 4.9); IR *γ*_max_ (KBr), 2924, 2854, 1738, 1621, 1460, 1260, 1064, 806, 721 cm^−1^; ^1^ H NMR (CDCl_3_), *δ* 5.36 (1 H, dd, *J* = 6.6, 7.2, H-3), 5.32 (1 H, m, H-4), 4.14 (2 H, d, *J* = 7.2 Hz), 2.78 (1 H, brm, w_1/2_ = 6.1 Hz, H-2β), 2.30 (2 H, m, H_2_-6), 2.05 (4 H, m, 2 × CH_2_), 1.59 (4 H, m, 2 × CH_2_), 1.28 (52 H, brs, 26 × CH_2_), 0.90 (3 H, t, *J* = 6.3, Me-37); ^13^ C NMR (CDCl_3_), *δ* 173.56 (C-1), 130.15 (C-3), 127.91 (C-4), 66.83 (C-5), 34.21 (C-2), 31.90 (CH_2_), 29.65 (21 × CH_2_), 29.33 (4 × CH_2_), 27.19 (CH_2_), 25.61 (CH_2_), 24.90 (CH_2_), 22.64 (CH_2_), 14.03 (Me-37); +ve ion FAB MS *m/z* (*rel. int.*), 546 [M]^+^ (C_37_H_70_O_2_) (19.7), 449 (31.8), 97 (71.0).

#### Hydroxy coriander lactone (2)

Elution of the column with chloroform afforded pale yellow crystals of **2**, recrystallized from acetone, 102 mg (0.12%yield); R_f_ value, 0.73 (chloroform); m.p., 95 to 96°C; UV *λ*_max_ (MeOH), 205 nm (log ϵ 5.1); IR *γ*_max_ (KBr), 3463, 2921, 2852, 1743, 1645, 1463, 1379, 1145, 1092, 1019, 722 cm^−1^; ^1^ H NMR (CDCl_3_), *δ* 5.34 (1 H, dd, *J* = 6.8, 7.5 Hz, H-3), 5.30 (1 H, m, H-4), 5.17 (1 H, brm, w_1/2_ = 8.5 Hz, H-26), 5.11 (1 H, brm, w_1/2_ = 9.5, H-27), 4.32 (1 H, d, *J* = 7.5 Hz, H-5a), 4.28 (1 H, d, *J* = 7.5 Hz, H_2_-5b), 4.16 (1 H, brm, w_1/2_ = 5.7 Hz, H-18β), *δ* 2.76 (1 H, m, w_1/2_ = 6.8 Hz, H-2β), 2.30 (2 H, m,H_2_-25), *δ* 2.02 (2 H, m, H_2_-28), 1.98 (2 H, m, CH_2_), 1.68 (2 H, m, CH_2_), 1.62 (2 H, m, CH_2_). 1.60 (2 H, m, CH_2_), 1.26 (50 H, brs, 25 × CH_2_), 0.87 (3 H, t, *J* = 6.5 Hz, Me-40); ^13^ C NMR (CDCl_3_), *δ* 172.78 (C-1), 130.34 (C-4), 129.78 (C-3), 128.78 (C-26), 127.96 (C-27), 68.83 (C-18), 65.95 (C-5), 42.03 (C-2), 33.76 (CH_2_), 31.79 (CH_2_), 31.68 (CH_2_), 29.54 (11 × CH_2_), 29.21 (5 × CH_2_), 29.02 (5 × CH_2_), 27.67 (CH_2_), 27.11 (CH_2_), 26.66 (CH_2_), 25.50 (CH_2_), 24.73 (CH_2_), 24.34 (CH_2_), 22.55 (CH_2_), 13.94 (Me-40); +ve ion FAB MS *m/z* (*rel. int.*), 603[M + H]^+^ (C_40_ H_75_ O_3_) (100), 559 (3.7), 503 (6.1), 461 (3.6), 405 (3.8), 393 (18.0), 337 (18.2), 295 (14.1), 265 (39.8), 97 (37.9).

#### Glyceryl-1,2-dioctadec-9,12-dienoate-3-octadec-9-enoate (3)

Elution of column with chloroform-methanol (19:1) afforded light green resinous mass of **3**, crystallized from acetone, 82 mg (0.096% yield); R_f_ value, 0.6 (chloroform-methanol, 19: 1); m.p., 65 to 66°C; UV *λ*_max_ (MeOH), 206 nm (log ϵ 4.6); IR *γ*_max_ (KBr), 2925, 2855, 1743, 17251640, 1460, 1375, 1167, 723 cm^−1^; ^1^ H NMR (CDCl_3_), *δ* 5.34 (4 H, m, H-9′, H-13′, H-9′′, H-13′′), 5.32 (4 H, m, H-10′, H-12′, H-10′′, H-12′′), 5.27 (2 H, m, H- 9′′′, H-10′′′), 4.33 (1 H, d, *J* = 1.7 Hz, H-2), 4.14 (2 H, m, H_2_-1), 4.11 (2 H, m, H_2_-3), 2.31 (1 H, d, *J* = 3.0 Hz, H_2_-2′a), *δ* 2.30 (1 H, d, *J* = 3.0 Hz, H_2_-2′b), 2.20 (1 H, d, *J* = 6.3 Hz, H_2_-2′′a), *δ* 2.18 (1 H, d, *J* = 6.3 Hz, H_2_-2′′b), 2.16 (1 H, d, *J* = 7.5 Hz, H_2_-2′′′a), 2.14 (1 H, d, *J* = 7.5 Hz, H_2_-2′′′b), 1.85 (2 H, m, H_2_-11′). 1.81 (2 H, m, H_2_-11′′), 1.79 (4 H, m, H_2_-8′, H-8′′), 1.77 (4 H, m, H2-14′, H_2_-14′′), 1.61 (4 H, m, H_2_-8′′′, H_2_-11′′′), 1.26 (48 H, brs, 24 × CH_2_), 1.16 (4 H, m, 2 × CH_2_), 0.87 (9 H, m, H_3_-18′, H_3_- 18′′, H_3_-18′′′); ^13^ C NMR (CDCl_3_) *δ* 173.37 (C-1′), 172.80 (C-1′′), 172.49 (C-1′′′), 130.22 (C-10′, C-10′′), 129.83 (C-12′), 129.66 (C-12′′), 128.68 (C-9′′′, C- 10′′′), 128.68 (C-9′, C-9′′) 127.82 (C-13′), 127.68 (C-13′′), 68.77 (C-2), 64.72 (C-1), 61.87 (C-3), 56.93 (C-2′), 56.62 (C-2′′), 56.31 (C-2′′′), 33.84 (C-11′), 33.81 (C-11′′), 31.68 (2 × CH_2_), 31.28 (CH_2_), 29.42 (10 × CH_2_), 29.09 (5 × CH_2_), 28.91 (5 × CH_2_), 27.54 (CH_2_), 26.96 (CH_2_), 26.55 (CH_2_), 25.94 (CH_2_), 25.38 (CH_2_), 28.58 (CH_2_), 24.23 (CH_2_), 22.42 (3 × CH_2_), 13.80 (Me-18′, Me-18′′, Me-18′′′); +ve ion FAB MS *m/z* (*rel. int.*), 880 [M]^+^ (C_57_ H_100_ O_6_) (1.5), 617 (29.8), 602 (37.0), 339 (71.8), 337 (31.1), 281 (21.5), 279 (31.6), 265 (24.8), 137 (26.2), 111 (39.9).

#### Glyceryl-1,2,3-trioctadecanoate (4)

Elution of column with chloroform-methanol (97: 3) furnished a colorless resinous mass of **4**, crystallized from from acetone, 132 mg (0.155% yielded); *R*_f_ value, 0.23(chloroform-methanol, 22: 3); m.p. 86 to 88°C; UV *λ*_max_ (MeOH), 207 nm (log ϵ 4.6); IR *γ*_max_ (KBr), 2924, 2854, 1725, 1721, 1459, 1373, 1265, 1170, 723 cm^−1^; ^1^ H NMR (DMSO-d6), *δ* 3.63 (1 H, m, H-2), 3.50 (2 H, m, H_2_-1), 3.23 (2 H, m, H_2_-3), 2.49 (2 H, brs, H_2_-2′′), 2.25 (2 H, brs, H_2_-2′), 2.18 (2 H, brs, H_2_-2′′′), 2.05 (2 H, m, CH_2_), 1.98 (4 H, m, 2 × CH_2_), 1.70 (2 H, m, CH_2_), 1.61 (4 H, m, 2 × CH_2_), 1.49 (6 H, m, 3 × CH_2_), 1.31 (8 H, brs, 4 × CH_2_), 1.22 (20 H, brs, 10 × CH_2_), 1.20 (18 H, brs, 9 × CH_2_), 1.18 (18 H, brs, 9 × CH_2_), 1.16 (8 H, brs, 4 × CH_2_), 0.84 (9 H, brs, 3 × CH_3_); ^13^ C NMR (DMSO-d6), *δ* 174.51 (C-1′′), 173.80 (C-1′), 172.72 (C-1′′′), 71.40 (C-2), 69.89 (C-1), 64.81 (C-3), 56.67 (C-1′′), 56.67 (C-1′), 51.35 (C-1′′′), 33.73 (CH_2_), 32.62 (CH_2_), 29.19 (20 × CH_2_), 28.87 (16 × CH_2_), 27.73 (CH_2_), 26.70 (CH_2_), 25.21 (CH_2_), 24.55 (CH_2_), 22.20 (3 × CH_2_); +ve ion FAB MS *m/z* (*rel. int.*), 890[M]^+^ (C_57_ H_110_ O_6_) (1.5), 283 (15.1), 267 (18.3).

#### n-nonadecanyl-n-docos-11-enoate (5)

Elution of column with chloroform-methanol (91:9) afforded light brown mass resinous mass of **5**, crystallized from acetone, 112 mg (0.131% yield); *R*_f_ value, 0.44 (chloroform-methanol, 22: 3); m.p., 71 to 73°C; UV *λ*_max_ (MeOH), 207 nm (log ϵ 4.3); IR *γ*_max_ (KBr), 2924, 2854, 1721, 1459, 1373, 1265, 1170, 723 cm^−1^; ^1^ H NMR (CDCl_3_), *δ* 5.34 (2 H, m, H-11. H-12), 3.79 (1 H, d, *J* = 9.9 Hz, H_2_-1′a), 3.75 (1 H, d, *J* = 9.9 Hz, H_2_-1′b), 2.62 (2 H, brs, H_2_-2), 2.30 (2 H, m, H_2_-10), 2.03 (2 H, m, H_2_-13), 1.60 (4 H, m, 2× CH_2_), 1.25 (60 H, brs, 30 × CH_2_), 0.87 (3 H, t, *J* = 6.3 Hz, Me-22), 0.84 (3 H, t, *J* = 6.1 Hz, Me-19′); ^13^ C NMR (CDCl_3_), *δ* 172.51 (C-1), 130.01 (C-11), 116.02 (C-12), 65.16 (C-1′), 51.74 (C-2), 32.88 (CH_2_), 31.76 (CH_2_), 29.53 (20 × CH_2_), 29.20 (9 × CH_2_), 27.07 (CH_2_), 24.53 (CH_2_), 22.52 (CH_2_), 13.94 (Me- 22, Me- 19′); +ve ion FAB MS *m/z* (*rel. int.*), 604[M]^+^ (C_41_ H_80_ O_2_) (23.1), 337 (31.0), 267 (22.5).

#### Oleiyl glycoside (6)

Elution of column with chloroform-methanol (22: 3) afforded colorless mass of **6**, crystallized from acetone, 145 mg (0.170% yield); *R*_f_ value, 0.2 (chloroform-methanol, 17: 3); m.p., 60 to 62°C; UV *λ*_max_ (MeOH), 209 nm (log ϵ 5.3); IR *γ*_max_ (KBr), 3410, 3360, 2925, 2855, 1733, 1640, 1457, 1261, 1091, 801 cm^−1^; ^1^ H NMR (CDCl_3_), *δ* 5.31 (2 H, m, H-9, H-10), 5.25 (1 H, brs, H-1′), 4.41 (1 H, m, H-5′), 4.19 (1 H, m, H-2′), 3.61 (1 H, m, H-3′), 3.58 (1 H, m, H-4′), 3.27 (2 H, brs, H_2_-6′), 2.67 (2 H, m, H_2_-2), 2.18 (2 H, m, H_2_-8), 1.93 (2 H, m, H_2_-11), 1.48 (4 H, brs, 2 × CH_2_), 1.16 (18 H, brs, 9 × CH_2_), 0.77 (3 H, t, *J* = 6.1 Hz, Me-18) . ^13^ C NMR (CDCl_3_), *δ* 173.67 (C-1), 129.85 (C-9), 127.71 (C-10), 103.15 (C-1′), 80.23 (C-5′), 69.83 (C- 2′), 68.06 (C-3′), 67.08 (C-4′), 61.05 (C-6′), 55.67 (C-2), 52.27 (CH_2_), 38.55 (CH_2_), 33.74 (CH_2_), 31.57 (CH_2_), 29.34 (3 × CH_2_), 29.02 (2 × CH_2_), 26.89 (CH_2_), 25.31 (CH_2_), 24.55 (CH_2_), 22.34 (CH_2_), 13.78 (Me-18); +ve ion FAB MS *m/z* (*rel. int.*), 445[M + H]^+^ (C_24_H_45_O_7_) (35.6), 265 (28.3), 180 (26.7).

## Conclusions

Phytochemical investigation of fruits of *C. sativum* led to isolate new aliphatic *δ*-lactones which may be used as chromatographic markers for quality control of the drugs.

## Competing interests

The authors declare that they have no competing interests.

## Supplementary Material

Additional file 1 Showing spectrum of ^1^H NMR and ^13^C NMR of coriander lactone and hydroxy coriander lactone and mass spectrum of hydroxy coriander lactone. The file contains ^1^H NMR, ^13^C NMR, and mass spectrum of coriander lactone and hydroxyl coriander lactone. Click here for file
