# Supplementary material for: Two new aliphatic lactones from the fruits of Coriandrum sativum L
Source: Org Med Chem Lett. 2012 Jul 16;2:28. doi: 10.1186/2191-2858-2-28 (PMC3434120; doi:10.1186/2191-2858-2-28)
Supplement: Additional file 1 — Showing spectrum of 1H NMR and 13C NMR of coriander lactone and hydroxy coriander lactone and mass spectrum of hydroxy coriander lactone. The file contains 1H NMR, 13C NMR, and mass spectrum of coriander lactone and hydroxyl coriander lactone. [file 2191-2858-2-28-S1.doc]

**
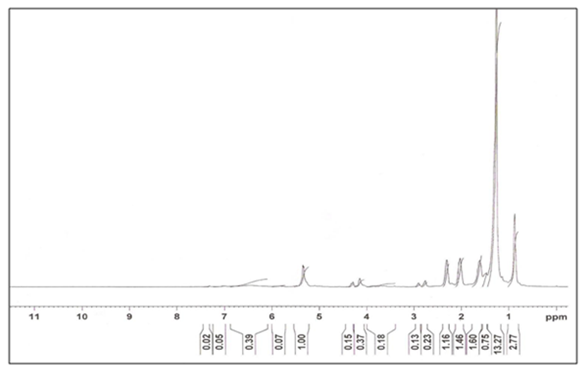
**

**Spectrum 1.1: ^1^H NMR spectrum of corianderolactone (1)**

**
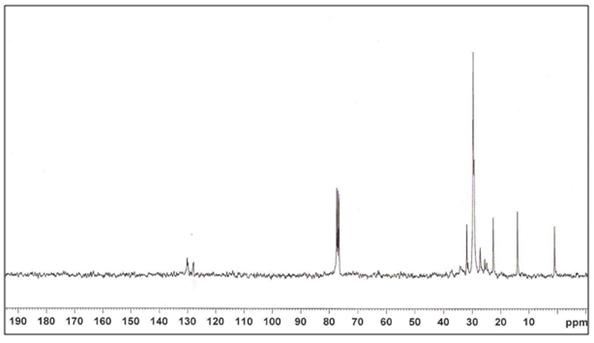
**

**Spectrum 1.2: ^13^C NMR spectrum of corianderolactone (1)**


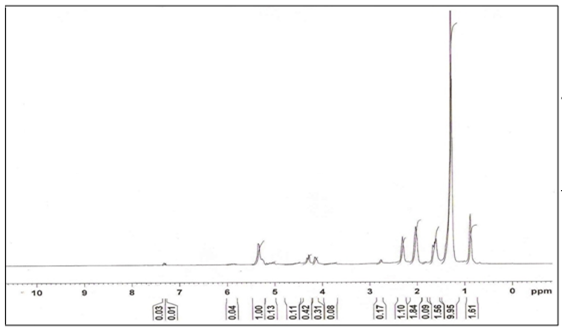


**Spectrum 1.3: ^1^H NMR spectrum of hydroxycorianderolactone (2)**


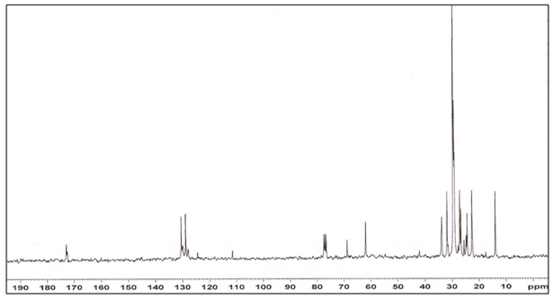


**Spectrum 1.4: ^13^C NMR spectrum of hydoxycorianderolactone (2)**


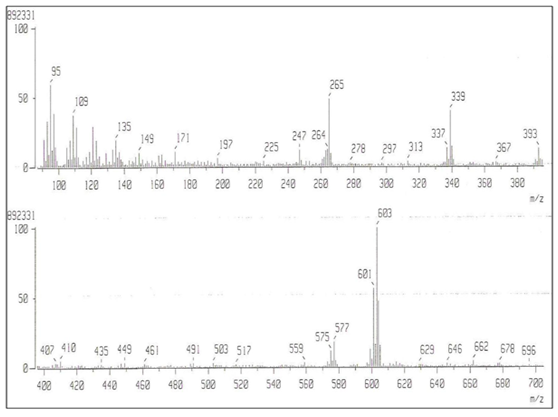


**Spectrum 1.5: Mass spectrum of hydroxycorianderolactone (2)**
